# Evolutionary diversification and immunoprofiling of cathepsin L toolkit in common carp

**DOI:** 10.3389/fcimb.2026.1805838

**Published:** 2026-04-07

**Authors:** Sagar Nayak, Jiří Kyslík, Jovana Majstorović, Tamás Dobai, Veronika Žánová, Hana Pecková, Tomáš Korytář, Pavla Bartošová-Sojková

**Affiliations:** 1 Institute of Parasitology, Biology Centre, Czech Academy of Sciences, České Budějovice, Czechia; 2 Faculty of Science, University of South Bohemia, České Budějovice, Czechia; 3 Faculty of Fisheries and Protection of Waters, University of South Bohemia, České Budějovice, Czechia

**Keywords:** cathepsin, gene expression, myxozoa, parasite challenge, phylogeny, *Sphaerospora molnari*, synteny, teleost immunity

## Abstract

Cysteine cathepsins are central regulators of lysosomal proteolysis, immune and metabolic homeostasis, yet the evolutionary forces shaping their diversification in vertebrates remain incompletely understood. Teleost fishes, particularly polyploid lineages, provide a powerful system to examine how whole-genome duplication remodels protease repertoires and regulatory networks. Here, using the polyploid common carp model, we identify nine cathepsin L (*ctsl)* paralogs retained after ancestral and lineage-specific whole-genome duplications, and show that these duplicates have undergone spatially distributed expression divergence. Paralog expression is strongly partitioned across tissues and immune compartments: *ctsl.1A/B* are enriched at mucosal barriers, whereas *ctslaA/B* predominate in metabolic and systemic immune hubs and are the only *ctsl* transcripts detected in circulating blood and head kidney leukocytes. Structural modelling predicts variation in protease loop architecture and active-site cleft geometry, suggesting divergent substrate preferences and/or catalytic properties. During infection with *Sphaerospora molnari*, a myxozoan pathogen, inflammatory cytokine induction is accompanied by transient *ctsl* downregulation, followed by a cell- and time-dependent reprogramming of proteolytic profiles as disease progresses. Collectively, these findings reveal a transcriptional pattern of *ctsl* regulation uncoupled from cytokine expression dynamics, emerging from retained *ctsl* duplicates. Moreover, they demonstrate how polyploidization can generate tissue- and cell-specific regulation of lysosomal pathways that balance host defense and homeostasis.

## 1 Introduction

Cysteine cathepsins of clan CA are lysosomal proteases that contribute to protein turnover, homeostatic signaling, and immune regulation across vertebrates, with additional roles in the cytoplasm, nucleus, and extracellular milieu (Barrett and Rawlings, 1996; Turk and Stoka, 2007; Brix et al., 2008). Within this group, cathepsin L (CTSL) is a multifunctional endolysosomal protease implicated, among others, in diverse immune processes, including antigen processing, regulation of inflammatory responses, leukocyte differentiation, and tissue remodeling (Nakagawa et al., 1998; Riese and Chapman, 2000; Uinuk-ool et al., 2003; Hou et al., 2015; Vizovišek et al., 2019; Li et al., 2022). In vertebrates, CTSL has diversified through gene duplication and positive selection, producing functionally distinct paralogs (Zhou et al., 2015). This is also evident in teleosts, where multiple paralogs display tissue-specific expression (Chen et al., 2011; Wang et al., 2015; Li et al., 2022), interact with pathogen-associated molecular patterns (Guan et al., 2022), and are upregulated during bacterial, fungal, and viral infections, highlighting roles in both systemic and mucosal immunity (Aranishi, 1999a; Aranishi, 1999b; Chen et al., 2020). This diversification reflects teleost genomic plasticity, driven by the teleost-specific third round of whole-genome duplication (Ts3R) (Glasauer and Neuhauss, 2014) and conserved synteny (Chen et al., 2020; Li et al., 2022). The common carp (*Cyprinus carpio*), an economically vital species (Balon, 1995; Bostock et al., 2016; Roy et al., 2020), underwent an additional carp-specific 4R duplication (Cs4R), further expanding the CTSL repertoire shared by polyploid cyprinids (Kolder et al., 2016; Yang et al., 2016; Kuhl et al., 2022; Wang et al., 2022; Chen et al., 2019; Lian et al., 2025).

While earlier bioinformatic and biochemical surveys identified multiple *ctsl* isoenzymes in common carp (Aranishi et al., 1997; Tsunemoto et al., 2004; Kolder et al., 2016), the lack of a systematic framework integrating evolutionary trajectories with spatiotemporal regulation leaves us without a clear map of how these enzymes are regulated during homeostatic and infection states.

Here, we decode the full *ctsl* repertoire across diverse common carp strains, through an integrated analysis of evolutionary background, syntenic collinearity with other cyprinids, and protease structural modeling. Additionally, we define the gene-specific transcriptional signatures across carp tissues and cell types during homeostasis to infer their potential involvement in proteolytic activities. Furthermore, to elucidate the involvement of *ctsl* in regulation of innate and adaptive immune responses we employed a previously published infection model of common carp with *Sphaerospora molnari* (Korytář et al., 2019; Dobai and Bartošová-Sojková, 2024). This myxozoan parasite, which causes severe systemic sphaerosporosis and anemia (Dyková et al., 1983; Eszterbauer et al., 2013), induces strong inflammatory and antibody responses in laboratory model and thus serves as an ideal model to elucidate the contribution of cathepsins to the immune responses (Korytář et al., 2019; Korytář et al., 2020). Altogether, our study presents an atlas of *ctsl* expression and evolution, providing a systems-level overview of the proteolytic regulation of innate and adaptive immunity in a complex host-parasite model.

## 2 Materials and methods

### 2.1 Phylogenomic identification of cyprinid *ctsl* repertoire

We implemented a comprehensive ortholog screening to systematically define the *ctsl* gene repertoire across the Cyprinidae family. Reference *ctsl* sequences from zebrafish (*Danio rerio)* were retrieved from the Ensembl (https://www.ensembl.org/index.html; accessed April 17, 2024) and ZFIN databases (http://zfin.org/; accessed April 17, 2024) and utilized as queries for tBLASTn searches against common carp (*Cyprinus carpio*) genomic assemblies (**Supplementary Table 1**; E-value cutoff 1 x 10^-5^). Candidate loci were cross-referenced with previously annotated common carp proteomic data (Kolder et al., 2016) and validated against the Ensembl repository (**Supplementary Table 2**). This strategy was extended to identify *ctsl* homologs in representative polyploid cyprinids, including goldfish (*Carassius auratus*), silver crucian carp (*Carassius gibelio*), and three *Sinocyclocheilus* barbel species (*S. grahami*, *S. rhinoceros*, *S. anshuiensis*) (**Supplementary Table 2**). To ensure evolutionary consistency, gene nomenclature was assigned based on strict orthology with zebrafish counterparts (**Table 1**), as corroborated by our subsequent phylogenetic reconstruction and micro-syntenic analysis.

**Table 1:** List of zebrafish *ctsl* genes and their orthologues in common carp A and B subgenomes.

Gene name in zebrafish	Gene symbol in zebrafish	Gene symbols of common carp orthologues
cathepsin L.1	*ctsl.1*	*ctsl.1A*; *ctsl.1B; ctsl.1B’*
cathepsin 12	*cts12*	*cts12A*; *cts12B*
cathepsin L-like	*ctsll*	*ctsllA*; *ctsllB*
cathepsin La	*ctsla*	*ctslaA*; *ctslaB*

The A and B carp *ctsl* genes are the result of carp-specific WGD (Cs4R), except *ctsl.1B’* that arose from *ctsl.1B* by tandem gene duplication.

### 2.2 Comparative genomics: synteny and collinearity analysis

By performing comparative synteny mapping across common carp, zebrafish, goldfish, silver crucian carp, and golden-line barbel, we assessed the macro-syntenic conservation of *ctsl* clusters and the structural integrity of their genomic neighborhoods across diploid and allotetraploid cyprinid lineages. Conserved genomic contexts and flanking gene neighborhoods were extracted from Genomicus (Nguyen et al., 2022) integrated with Ensembl, and supplemented with Ensembl Rapid Release data for the silver crucian carp. For common carp, micro-syntenic blocks were refined using the chromosome-level assembly by Li et al. (2021) to ensure maximum positional accuracy. Genome-wide collinearity between *C. carpio* and related cyprinids was quantified using MCScanX (Wang et al., 2024), employing a stringent scoring matrix match score: 50; gap penalty: –1; overlap window: 5 and a statistical threshold: E < 10^-10^ for collinear gene pairs. To distinguish true orthologs from lineage-specific duplicates, syntenic evidence was cross-validated against our phylogenetic reconstructions. High-resolution macro-syntenic relationships and chromosomal rearrangements were visualized via Circos plots generated using TBtools-II (Chen et al., 2023), providing a comprehensive map of the orthologous landscape across the cyprinid radiation.

### 2.3 Phylogenetic reconstruction of cyprinid *ctsl* genes

To resolve the evolutionary history of fish *ctsl* genes, amino acid sequences were retrieved from GenBank (search term: “cathepsin L”) and integrated with homologs identified from the cyprinid genomic data described above (see section 2.1). This yielded a comprehensive dataset of 808 sequences. Multiple sequence alignment was performed using MAFFT v7.017 within the Geneious Prime v2019.0.4 framework (Kearse et al., 2012). The alignment was subsequently inspected manually in Geneious Prime, and ambiguously aligned or excessively gap-rich positions were removed prior to phylogenetic analysis, resulting in a final alignment of 412 amino acid positions. To achieve higher resolution for specific clades, targeted subsets were constructed for *ctsl.1, ctsla, cts12*, and *ctsll* homologs, including representatives of non-cyprinid species as outgroups. Maximum likelihood phylogenies were inferred with RAxML-NG (Kozlov et al., 2019) under the WAG+G model. The amino-acid substitution model was selected beforehand using ModelFinder (Kalyaanamoorthy et al., 2017) as implemented in IQ-TREE (Nguyen et al., 2015), which evaluated candidate models and, under default settings, selected the best-fit model by BIC. Branch support was assessed with 500 bootstrap replicates for the large dataset and 1,000 replicates for the subset alignments. Fish were classified following established phylogenetic standards (Betancur-R et al., 2017). Fish silhouettes were sourced from PhyloPic v2.0 (www.phylopic.org).

### 2.4 Fish husbandry and animal handling

All animal experiments were conducted in accordance with European Union Directive 2010/63/EU and the relevant Czech legislation (Section 29 of Act no. 246/1992 Coll. on the Protection of animals against cruelty, amended by Act no. 77/2004 Coll.). Experimental protocols were reviewed and approved by the Ethics Committee of the Czech Academy of Sciences (protocols No. 88/2019 and 116/2023-P). Specific-pathogen-free (SPF) European common carp were maintained in the recirculating aquaculture system (RAS) at the Institute of Parasitology, Biology Centre CAS, following established protocols. Fish were kept under a stable 12-hour light/dark cycle and fed a commercial diet daily. Water quality parameters, including temperature, dissolved oxygen, and nitrogenous waste levels, were monitored regularly to ensure optimal health as previously described (Korytář et al., 2019; Holzer and Pimentel Santos Da Conceição, 2021). Fish were deeply anesthetized by overdose immersion in clove oil (0.6 mL/L) for blood collection and euthanasia was completed by decapitation prior to tissue sampling.

### 2.5 Tissue collection and sample preparation

To validate bioinformatically identified carp *ctsl* genes and assess basal expression profiles, 14 tissues and cell types were harvested from three SPF common carp. Collected samples included the gills, intestine, skin, spleen, muscle, head kidney, head kidney leukocytes, brain, trunk kidney, liver, testes, thymus, medulla oblongata, and whole blood. Peripheral blood was drawn from the caudal vein using heparinized syringes as previously described (Holzer and Pimentel Santos Da Conceição, 2021). Head kidney leukocytes were isolated following Montero et al. (2019). Briefly, kidney tissue was homogenized through 100 µm strainers (Corning, USA) in RPMI 1640 medium (Gibco, UK), washed, and layered onto 25% Percoll and centrifuged at 500 × g for 10 min. The resulting cell pellet was washed and resuspended in RNAprotect Cell Reagent (Qiagen, Germany), incubated overnight at 4 °C, and stored at -80 °C until processing. All other samples were preserved and stored under the same conditions.

### 2.6 RNA extraction, cDNA synthesis, and gene verification

Total RNA was extracted from RNAprotect-preserved samples with the RNeasy Mini Kit (Qiagen, Germany). RNA concentration and purity were assessed using Nanodrop ND1000 (Thermo Fisher Scientific, USA). To eliminate genomic DNA contamination, 500 ng of total RNA was treated with gDNA Wipeout buffer supplemented in QuantiTect Reverse Transcription kit (Qiagen, Germany) prior to reverse transcription. First-strand cDNA synthesis was performed using the QuantiTect Reverse Transcription Kit (Qiagen, Germany) at 42 °C for 30 min, followed by 95 °C for 5 min. The *in silico* identified *ctsl* sequences were validated via PCR using the WizPure HS-PCR FDmix (Wizbiosolutions Inc., Republic of Korea) and gene-specific primers (**Supplementary Table 3**). The amplification protocol consisted of 95 °C for 3 min; 30 cycles of 94 °C for 1 min, 52–60 °C for 1 min, 72 °C for 2 min; and a final extension at 72 °C for 10 min. PCR products were purified using the Genepure kit (Geneaid Biotech Ltd., Taiwan) and verified by Sanger sequencing (SEQme, Czech Republic). Comprehensive details regarding primer sequences, optimized annealing temperatures, and amplicon characteristics are provided in **Supplementary Table 3**.

### 2.7 Protein structural modelling and motif analysis

Signal peptides for each common carp *ctsl* sequence identified for structural analysis (**Supplementary Data Sheet 1**) were predicted with the SignalP6.0 webserver (Teufel et al., 2022) and removed prior to modeling. As we aimed to compare the differences within the carp *ctsl* repertoire, structures of procathepsin Ls (zymogen, immature enzyme) were predicted using the Alphafold 3 webserver (Abramson et al., 2024). Using procathepsin L structures allows for the assessment of maximal structural differences as primary variations among cathepsin L enzymes are localized within their regulatory proregions (Turk et al., 2012). For each *ctsl* sequence, the highest-ranked model was selected based on pLDDT and PAE scores and visualized in Maestro (Release 2025-3: Schrödinger LLC, USA). Models were processed with Maestro’s Protein Preparation Wizard and superposed on backbone amides. Alignments were visually inspected for canonical cysteine peptidase motifs at the active site cleft. Motif-containing regions were further visualized and aligned in Maestro’s Multiple Sequence Viewer.

### 2.8 Parasite purification and fish challenge experiments

*S. molnari* blood stages (SMBS) were obtained from the common carp-*S. molnari* host-parasite acquisition model (Holzer and Pimentel Santos Da Conceição, 2021; Born-Torrijos et al., 2022). Pure SMBS fractions were isolated from carp blood cells using DEAE-cellulose ion-exchange chromatography and quantified in a Bürker hemocytometer. SPF carp were injected intraperitoneally with 10^6^ SMBS in 100 µL PBS, while control fish received PBS only (Montero et al., 2019; Born-Torrijos et al., 2022). Samples were collected at early infection stages (1, 3, 7 days post infection (dpi)) and during the known peak infection in the blood (28 dpi) and liver (42 dpi) (Korytář et al., 2019). At each time point, three fish were euthanized, and tissues (gills, intestine, spleen, head kidney, trunk kidney, and liver) were preserved in RNAprotect Cell Reagent (Qiagen, Germany), incubated overnight at 4 °C, and stored at -80 °C. Peripheral blood was collected from the caudal vein, diluted 1:2 in ice-cold RPMI 1640 (Gibco, UK), and fractionated on 3 mL Ficoll-Paque™ (Cytiva, Sweden) by centrifugation (500 × g, 10 min, 4 °C) to isolate leukocytes and erythrocytes, which were washed and stored in RNAprotect Cell Reagent (Qiagen, Germany).

### 2.9 qPCR and gene expression analysis of carp cathepsins and cytokines

Gene expression of *ctsl* and cytokine genes was analyzed using qRT-PCR with two distinct chemistries. RNA extraction and cDNA synthesis were performed as described in section 2.6. All reactions were run on a QuantStudio™ 6 Flex System (Applied Biosystems, USA) with technical triplicates, including no-template controls (NTC). Discrepancies between replicates (>0.5 Ct) were addressed by repeating reactions, adjusting for inter-run calibrators when necessary, and retaining only measurements with low standard deviations. For *ctsl* genes, a duplex TaqMan assay was employed with *β-actin* as the housekeeping gene. Primers and dual-labeled 5′-HEX/3’-BHQ1 TaqMan probes were designed for *ctsl* genes (**Supplementary Table 4**), while published oligonucleotides were used for *β-actin* (Korytář et al., 2019). Reactions (25 µL) contained 1× LightCycler^®^ 480 Probes Master (Roche, Switzerland), 0.4 µM primers, 0.2 µM probes, 5 µL cDNA, and PCR-grade water. The cycling program was: 95 °C for 30 s, followed by 40 cycles of 95 °C for 15 s and 60 °C for 1 min. Basal expression was calculated relative to the tissue with the highest signal (set to 100) and normalized to *β-actin*. Expression of the duplicated *ctsl.1B′* gene could not be assessed due to high sequence similarity with *ctsl.1B* preventing gene-specific primer design. Infection-induced expression changes of *ctsl.1A*/*B* and *ctslaA*/*B* were determined using the 2^-ΔΔCt^ method relative to PBS-injected controls. For cytokine genes (*tnfα*, *il-1β*, *il10*, *il6*, *ifnγ*), SYBR Green-based qRT-PCR was performed. Reactions (20 µL) contained 2 μL of 10–20-fold diluted cDNA, 10 μL Fast SYBR Green Master Mix (Applied Biosystems, USA), and 0.4 μM of each primer (**Supplementary Table 5**). The cycling program was: 95 °C for 20 s, followed by 40 cycles of 95 °C for 1 s and 60 °C for 20 s. Expression was normalized to the geometric mean of two housekeeping genes (*ef1-α* and *β-actin*) and analyzed using the Pfaffl method (Pfaffl, 2001), with fold changes expressed relative to uninfected controls.

### 2.10 Statistical analyses and data visualization

Statistical analyses were performed using GraphPad Prism 8 (GraphPad Software, San Diego, CA, USA). Differences between control and infected samples were assessed using unpaired *t*-tests, with multiple comparisons corrected by the Holm–Šidák method and significance level set at α < 0.05. Expression changes across time points were evaluated by one-way ANOVA followed by Tukey’s *post hoc* test. All heatmaps of Log2 fold change (FC) gene expression were generated in R (R Core Team, 2017) using the ggplot2 package (Wickham, 2016) and Heatmapper2 (Babicki et al., 2016). Principal component analysis (PCA) of *ctsl* and cytokine gene expression data was performed using the prcomp function in R (version 4.3.0). Confidence ellipses were generated at 95% to visualize tissue-specific clustering patterns. Data visualization was performed using the ggplot2 package (version 3.4.0). Pearson correlation analyses were performed using Morpheus software (https://software.broadinstitute.org/morpheus).

## 3 Results

### 3.1 Genomic landscape of *ctsl* gene repertoire in cyprinids

Systematic orthology screenings across seven examined common carp strains (**Supplementary Table 1**) identified a conserved repertoire of nine *ctsl* paralogs: *ctsl.1A/B/B′, ctsllA/B, ctslaA/B*, and *cts12A*/*B* (**Supplementary Table 2**). This expanded gene set is strictly maintained across allopolyploid cyprinids, including goldfish, silver crucian carp, barbels, contrasting with the fourth-ortholog baseline found in the diploid zebrafish (**Supplementary Table 2**). Detailed chromosomal mapping and sequence characteristics are listed in **Supplementary Table 6**.

### 3.2 Evolutionary trajectory and WGD-driven diversification of *ctsl*

Phylogenetic reconstruction resolved three ancestral clades, *ctsl.1*, *cts12*, and *ctsla*, rooted by early chordate (lancelet) and agnathan (lamprey/hagfish) homologs (**Supplementary Figure 1**). The *ctsla* clade further included the Cypriniformes-specific *ctsll* subclade. Paralogous distribution across gnathostomes aligns with early vertebrate (1R) and gnathostome (2R) whole-genome duplications (WGD) (Yu et al., 2024), followed by selective lineage-specific losses. Within *ctsl.1* and *cts12* clades, basal branching patterns track the transition from chondrichthyans through non-teleost actinopterygians to the otophysan radiation. While most teleosts maintain single-copy representation in these clades, notable expansions in *cts12* were detected in chondrichthyans and acipenserids (**Supplementary Figure 1**), suggesting recurrent, independent gene specialization. The *ctsla* clade, however, showed a complex evolutionary trajectory defined by asymmetric retention of Ts3R-derived paralogs. While most Clupeocephala lineages underwent reductive evolution to a single *ctsla* copy, Cypriniformes uniquely retained and diverged the second Ts3R-paralog, giving rise to the *ctsll* gene (**Supplementary Figure 1**).

**Figure 1:**
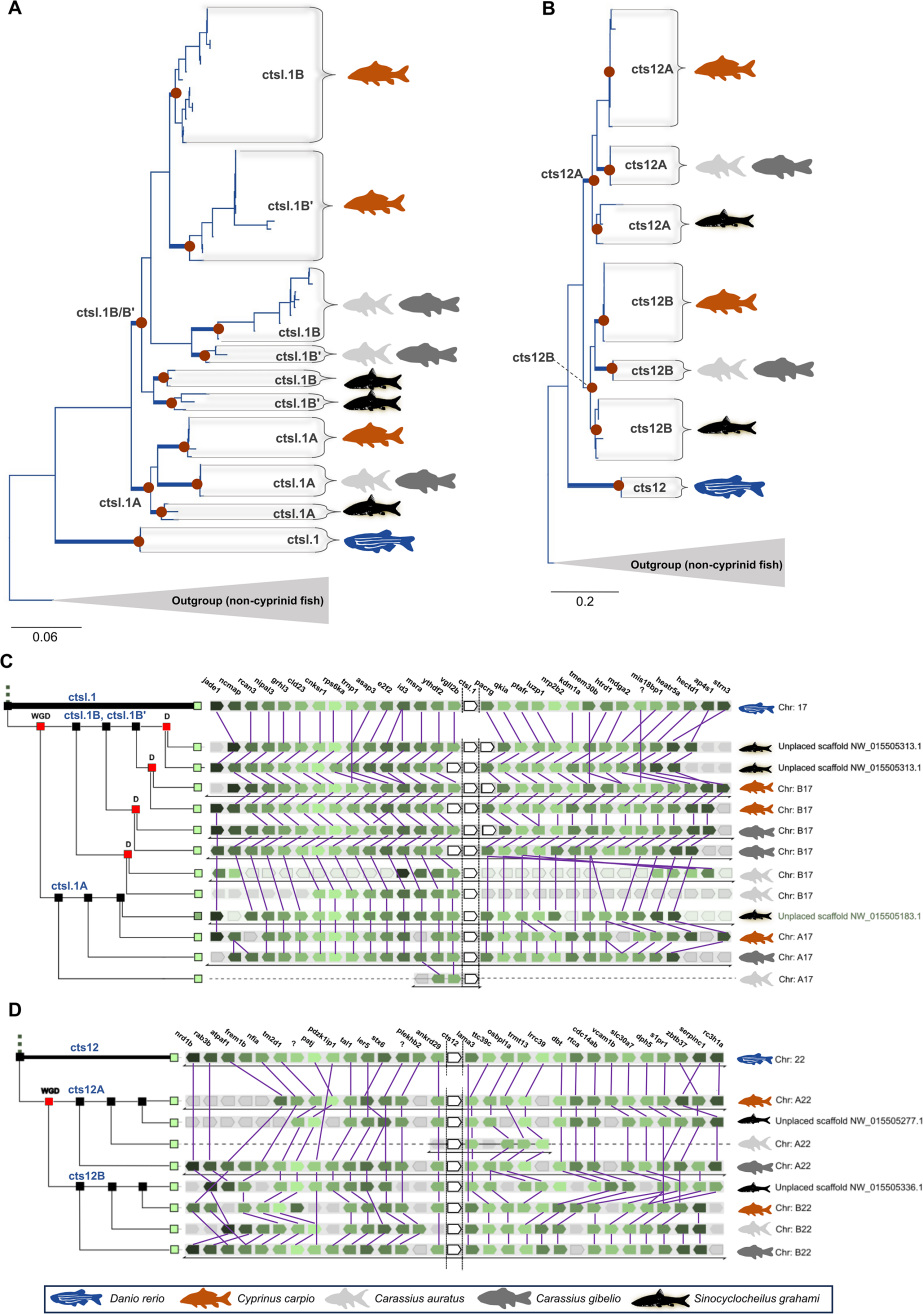
Evolutionary trajectory and micro-syntenic map of cyprinid ctsl.1 and cts12 lineages. **(A, B)** Maximum likelihood phylogenetic reconstruction of cyprinid *ctsl.1***(A)** and *cts12***(B)** genes. Trees include homologs from allopolyploid cyprinids (*Cyprinus carpio*, *Carassius auratus*, *C. gibelio*, and *Sinocyclocheilus grahami*), with the diploid *Danio rerio* as a reference. Non-cyprinid teleost *ctsl* homologs were used as outgroups. Brown circles denote nodes of individual cathepsin subclades (refer to **Supplementary Figure 1** for the full vertebrate topology and **Supplementary Figure 2** for the original cyprinid cathepsin trees). **(C, D)** Conserved micro-synteny maps of *ctsl.1***(C)** and *cts12***(D)** loci (white boxes). Syntenic blocks illustrate the high degree of collinearity across cyprinid genomes, where orthologous pairs reside on homologous chromosomes derived from the Cs4R (WGD). Distinct gene duplication events **(D)** are highlighted where lineage-specific expansions occurred. Plotted species are defined in the legend; full gene nomenclature is provided in **Table 1**. Fish silhouettes were obtained from PhyloPic v2.0 (www.phylopic.org).

**Figure 2:**
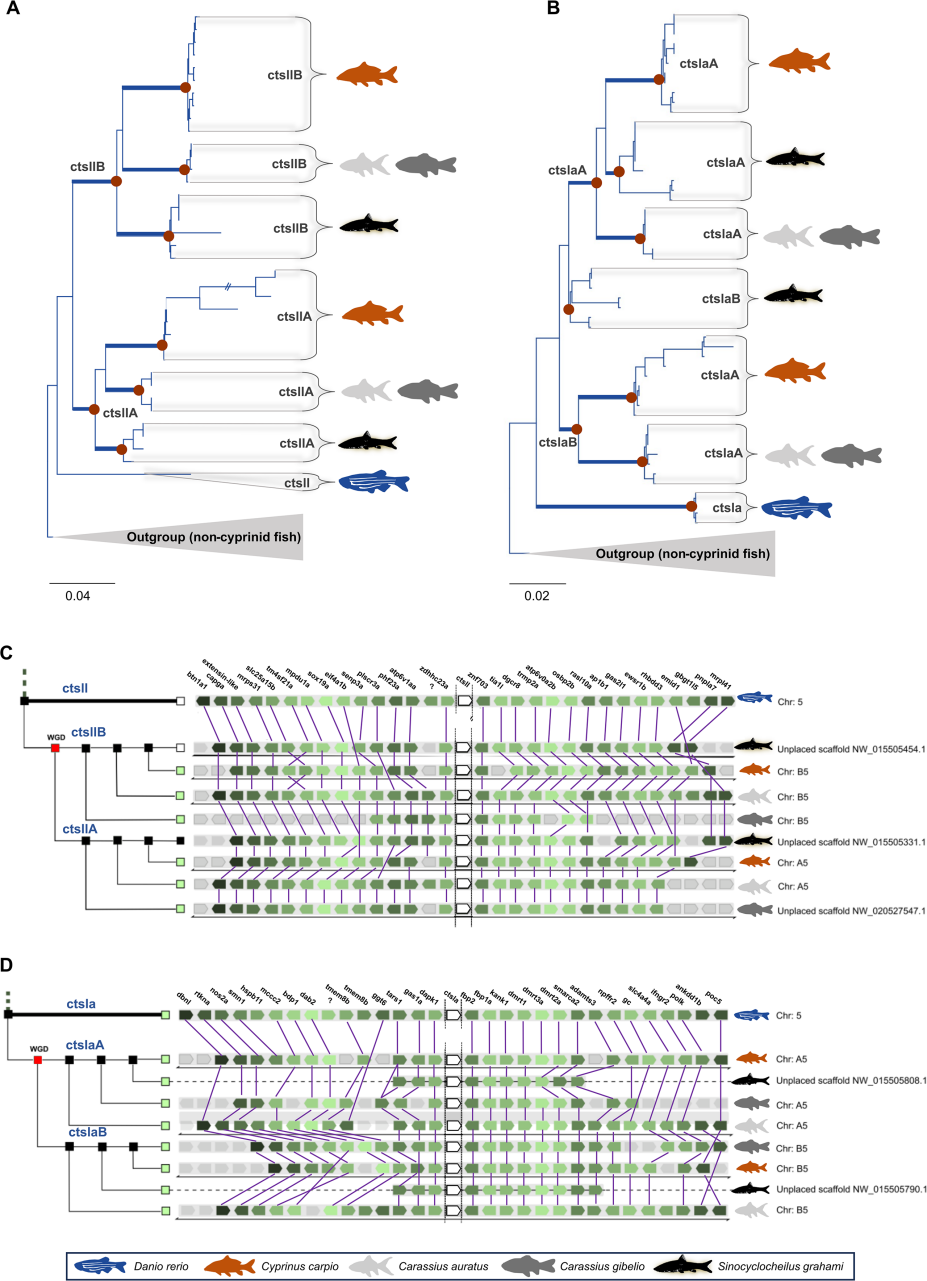
Evolutionary trajectory and micro-syntenic map of cyprinid ctsll and ctsla lineages. **(A, B)** Maximum likelihood phylogenetic reconstruction of cyprinid *ctsll***(A)** and *ctsla***(B)** genes. Trees include homologs from allopolyploid cyprinids (*Cyprinus carpio*, *Carassius auratus*, *C. gibelio*, and *Sinocyclocheilus grahami*), with the diploid *Danio rerio* as a reference. Non-cyprinid teleost *ctsl* homologs were used as outgroups. Brown circles denote nodes of individual cathepsin subclades (refer to **Supplementary Figure 1** for the full vertebrate topology and S2 for the original cyprinid cathepsin trees). **(C, D)** Conserved micro-synteny maps of the *ctsll***(C)** and *ctsla***(D)** loci (white boxes). Syntenic blocks illustrate the high degree of collinearity across cyprinid genomes, where orthologous pairs reside on homologous chromosomes derived from the Cs4R (WGD). Plotted species are defined in the legend; full gene nomenclature is provided in **Table 1**. Fish silhouettes were obtained from PhyloPic v2.0 (www.phylopic.org).

**Figure 3:**
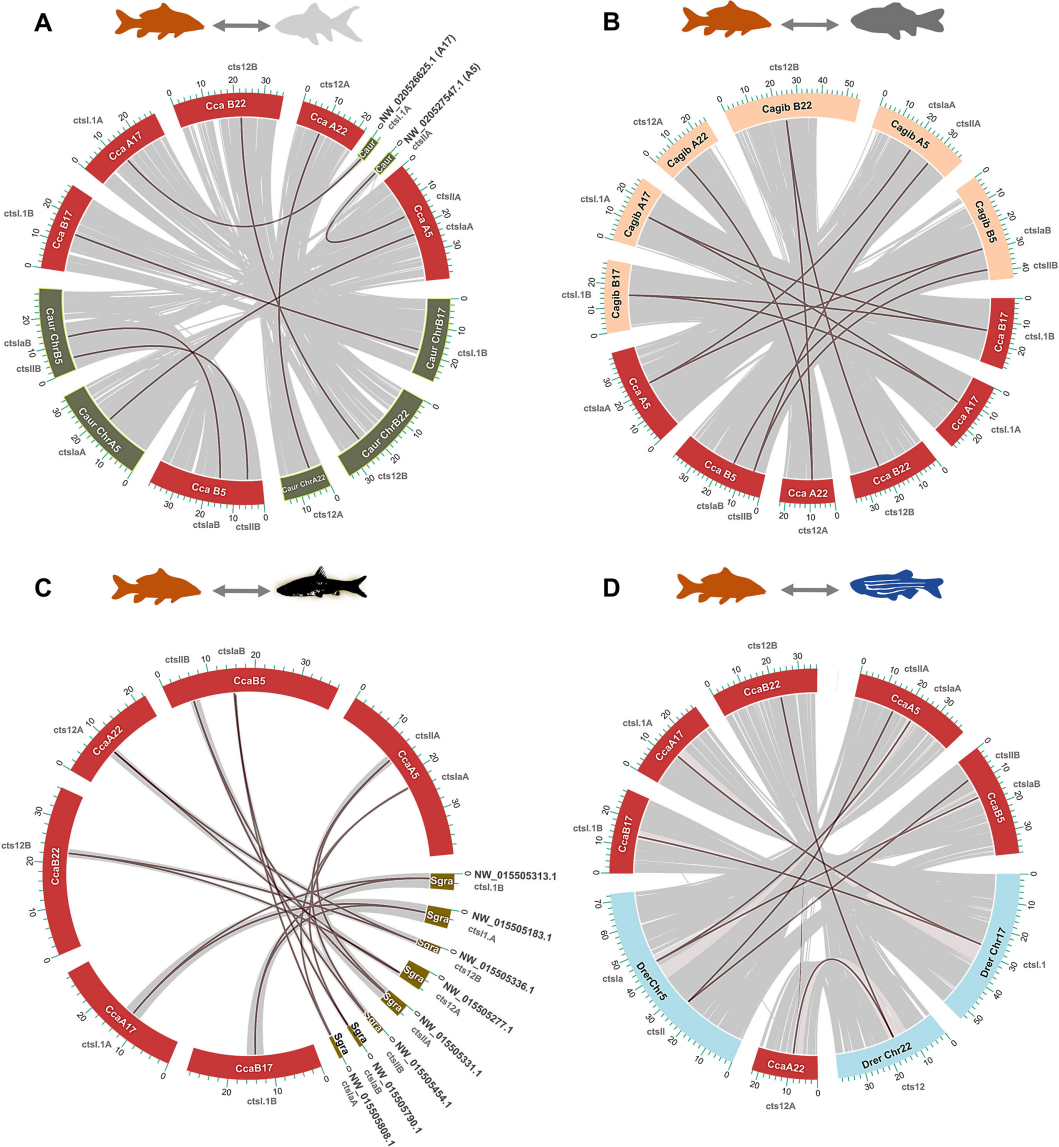
Macro-syntenic conservation and chromosomal collinearity of *ctsl* loci across the Cyprinidae radiation. Circos reconstructions illustrate the high-resolution collinearity between *Cyprinus carpio* (Cca) and representative cyprinids: **(A)***Carassius auratus* (Caur), **(B)***Carassius gibelio* (Cagib), **(C)***Sinocyclocheilus grahami* (Sgra), and **(D)***Danio rerio* (Drer). The common carp chromosomes harboring *ctsl* clusters are highlighted in red, with corresponding homologous segments in the compared species color-coded (green, light orange, dark green, or light blue). Gray ribbons delineate conserved syntenic blocks, while red synteny lines highlight the direct orthologous relationships and positional conservation of *ctsl* genes. These maps demonstrate the structural stability of the *ctsl* genomic landscape throughout the diverse polyploidization events of the cyprinid lineage.

Within the Cyprinidae, common carp *ctsl* sequences partitioned into four distinct sub-clades (*ctsl.1*, *cts12, ctsla*, and *ctsll*) with zebrafish orthologs consistently occupying basal, unduplicated positions (**Supplementary Figures 1**, **2**). The appearance of A and B subclades in polyploid cyprinids directly reflects Cs4R (**Figures 1A, B**; **Figures 2A, B**). Furthermore, the lineage-specific tandem duplication of *ctsl.1B* into *ctsl.1B′* (**Figure 1A**) underscores an ongoing, dynamic expansion of the proteolytic toolkit. Consistent with these findings, conserved micro-synteny (**Figures 1C, D**, **2C, D**, **3**) confirms that the diversity of the common carp *ctsl* family is a cumulative product of ancient vertebrate WGDs, teleost-specific 3R, and recent group-specific polyploidization events. Syntenic analyses reveal a high degree of macro-syntenic conservation and collinearity of *ctsl* gene clusters across the Cyprinidae family, reflecting a stable genomic architecture maintained since the common ancestor of these lineages (**Figure 3**). Comparisons with the allotetraploids *C. auratus* and *C. gibelio* (**Figures 3A, B**) demonstrate near-perfect subgenomic preservation, with *ctsl* loci anchored on homeologous chromosomes (e.g., Cca A5/B5 and A17/B17), underscoring the structural integrity of the genome post allotetraploidization. Despite assembly fragmentation in *S. grahami* (**Figure 3C**), micro-syntenic blocks confirm that the local gene-order motifs are strictly conserved (**Figures 1**, **2**). In contrast, mapping against the diploid *D. rerio* (**Figure 3D**) illustrates a characteristic 2n syntenic expansion, identifying *Drer* chromosomes 5, 17, and 22 as the ancestral templates for the expanded *ctsl* repertoire in *C. carpio*.

### 3.3 Structural diversity in the common carp CTSL homologs

**Figure 4:**
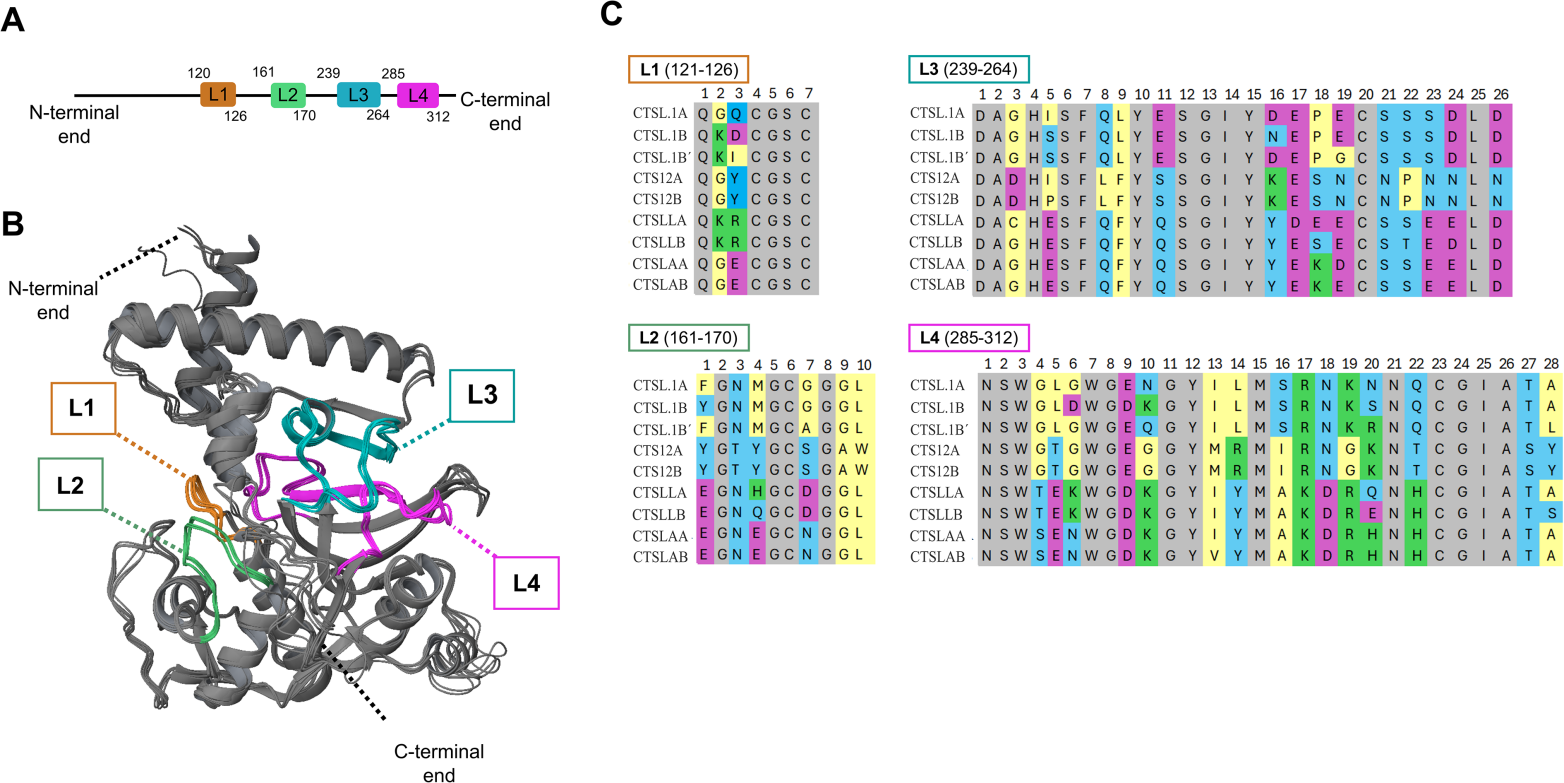
AlphaFold 3-based structural modeling and comparison of loops in the active-site cleft of common carp procathepsin Ls. **(A)** Representative domain architecture model of procathepsin L (CTSL), highlighting four active site loops: loop 1 (L1, orange), loop 2 (L2, green), loop 3 (L3, cyan), and loop 4 (L4, magenta). **(B)** Three-dimensional structural alignment of all CTSL paralogs. The four highly variable loops that delineate the substrate-binding cleft are color-coded, following the same colour arrangement as in section **(A)**. **(C)** Multiple sequence alignments of loops L1–L4 across the nine carp CTSL paralogs. Residues conserved across all nine CTSL sequences are shaded gray; hydrophobic residues are yellow; hydrophilic residues are light blue; negatively charged residues are magenta; and positively charged residues are green. GenBank identifiers: CTSL.1A = XP_042630078; CTSL.1B = XP_042598337; CTSL.1B’ = XP_042598339; CTS12A = XP_042567697; CTS12B = XP_042605645; CTSLLA = KTG04926; CTSLLB = XP_042579765; CTSLAA = XP_018956035; CTSLAB = XP_018953415.

To evaluate whether the nine carp *ctsl* paralogs encode structural differences suggestive of functional specialization, we generated and compared predicted zymogen models with particular emphasis on the active-site cleft loops (L1–L4), which shape substrate recognition and catalytic microenvironment (**Figure 4A**). All models exhibited high topological fidelity (pLDDT > 90), with the lowest confidence scores isolated to distal termini, whereas the L1–L4 maintained exceptionally high local confidence scores. High-resolution backbone superposition yielded pairwise root-mean-square deviation (RMSDs) < 1.5 Å, confirming a strictly conserved global fold (**Figure 4B**). While these models represent zymogens, the core α/β scaffold and active-site cleft architecture are pre-configured in the pro-protein and remain structurally invariant upon maturation, a hallmark of papain-like proteases. Consequently, the sequence-level divergence observed in L1–L4 dictates functional differences, as these loops determine the specific properties of the mature substrate-binding pocket (**Figure 4C**). Comparative analysis revealed marked intergroup biochemical heterogeneity despite global fold conservation. In L1, *ctsla* homologs harbor an acidic-hydrophobic motif, whereas *ctsll* sequences possess a strongly electropositive signature (**Figure 4C**).

### 3.4 Spatial and cellular partitioning of *ctsl* repertoire across mucosal and systemic niches

**Figure 5:**
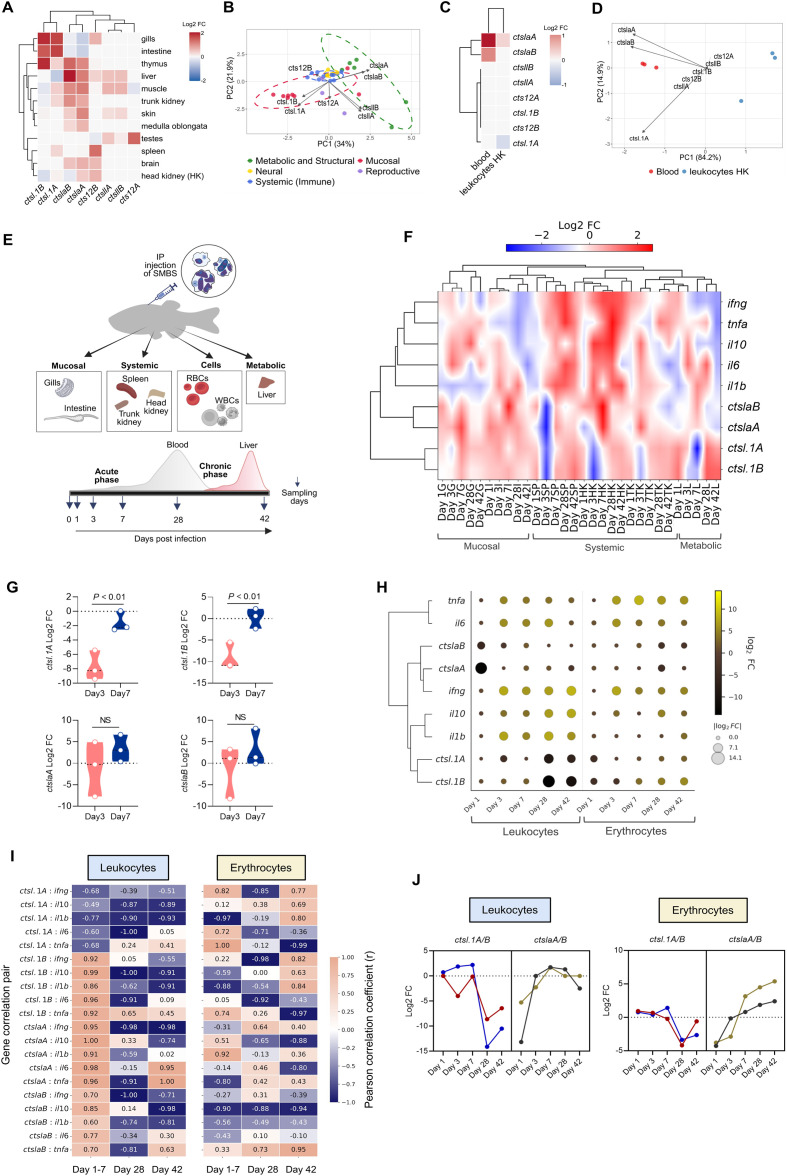
Spatiotemporal expression dynamics and immune signatures of cathepsin L transcripts in common carp during *Sphaerospora molnari* infection. **(A)** Clustered heatmap of constitutive cathepsin L (*ctsl*) transcript expression across various tissues of naïve common carp (*Cyprinus carpio*); data are normalized to the mean expression for each transcript (Log2 Fold Changes (FC)). **(B)** Principal Component Analysis (PCA) of tissue-specific *ctsl* expression. Data points are colored by functional clusters: systemic immune, mucosal defense, metabolic/structural, reproductive, and neural. Ellipses represent a 95% confidence interval. Vectors indicate the influence of individual *ctsl* genes on the sample distribution. **(C)** Clustered heatmap of constitutive *ctsl* expression profiles in peripheral blood and head kidney (HK) leukocytes. Data are normalized to the mean expression for each transcript (Log2 FC). **(D)** PCA of cellular-based *ctsl* expression showing distinct clustering of hematopoietic and immune cell lineages. **(E)** Experimental design of the *S. molnari* infection model, illustrating intraperitoneal injection (IP) of parasite blood stages (SMBS) and gradual sampling of systemic immune, mucosal tissues, immune cells, and metabolic organs over 42 days. **(F)** Hierarchical clustering heatmap of Log2 FC for representative *ctsl* and cytokine genes across systemic, mucosal, and metabolic tissues during infection. Abbreviations for tissues are: G, gills; I, intestine; SP, spleen; HK, head kidney; TK, trunk kidney; L, liver. **(G)** Comparison of acute-phase responses at day 3 versus day 7 post-infection, showing significant upregulation of specific *ctsl* isoforms (*ctsl.1A/B* - top; *ctslaA/B* - bottom) at day 7 (*P* < 0.01, n = 3; NS, not significant). Data were statistically analyzed using an unpaired t-test. **(H)** Clustered dot plot of Log2 FC expression for *ctsl* genes and cytokines in leukocytes and erythrocytes; dot radius represents the scale of fold change, and color intensity represents scaled expression values. **(I)** Pearson correlation heatmaps of *ctsl* gene variants and inflammatory cytokines in leukocytes (left) and erythrocytes (right) across a 42-day infection time course. Values represent correlation coefficients ranging from -1.0 (strong negative correlation, dark blue) to +1.0 (strong positive correlation, salmon/orange). Time points are indicated as days post-infection. **(J)** Temporal expressional profiling of *ctsl.1A/B* and *ctslaA/B* cathepsin transcripts in leukocyte and erythrocyte populations over 42 days post parasite challenge.

Expressional profiling of naïve carp revealed clear spatial and cellular differences in the distribution of *ctsl* paralogs across mucosal, systemic, and metabolic tissues and cells. Consistent with the paleotetraploid origin of carp genome, individual *ctsl* genes displayed distinct expression patterns, in line with data extracted from the previously published carp whole-body transcriptomic profiling (**Supplementary Figure 3**). Our analysis of 12 tissues showed that while *ctsl.1A/B* transcripts were predominantly expressed in mucosal sites (gills and intestine), the *ctslaA/B* cluster was constitutively expressed within systemic immune and metabolic niches, with the highest expression in liver (**Figure 5A**). This regulatory landscape is further defined by divergence of the *cts12A* paralog, which exhibits a testes-restricted expression profile distinct from the broader *cts12B*, *ctsl.1A/B*, *ctslaA/B*, and *ctsllA/B* clades. Such spatial distribution, validated by PCA clustering (**Figure 5B**), underscores the evolutionary success of gene duplication in tailoring lysosomal proteases to the specific physiological demands of mucosal and systemic compartments. To bridge the transcriptional landscape with cellular state, we profiled cells within total blood and head kidney leukocytes (**Figures 5C, D**), where only *ctslaA/B* paralogs showed detectable expression while other paralogs were not detected.

### 3.5 Temporal asynchrony between *ctsl* regulation and the inflammatory response

To investigate spatiotemporal regulation following *S. molnari* challenge (**Figure 5E**), we profiled four *ctsl* paralogs (*ctsl.1A, ctsl.1B, ctslaA, ctslaB*) characterized by high basal expression in immune-relevant and parasite-targeted tissues, including the blood, gills, and liver (**Figures 5A–C**). This infection triggered a profound, tissue-specific realignment of the *ctsl* landscape and canonical inflammatory signaling. Hierarchical clustering (**Figure 5F**) identified a distinct regulatory split, where a tight co-regulatory trajectory of *ctsl* transcripts appeared dissociated from hallmark pro- and anti-inflammatory cytokines (**Figure 5F**; **Supplementary Figures 3**, **5A**, **8A**). Within the systemic immune hubs (head kidney and spleen), a robust inflammatory signature emerged primarily during the chronic phase (28–42 dpi) (**Figure 5F**, **Supplementary Figure 6A**). Notably, this was preceded by an acute-phase suppression of cathepsin transcripts across systemic and metabolic compartments at 3 dpi, followed by a compensatory upregulation by 7 dpi (**Figures 5F, G**).

### 3.6 Divergent erythrocyte-leukocyte kinetics and the sequential realignment of *ctsl* during *S. molnari* infection

Longitudinal profiling of blood fractions revealed that leukocytes and erythrocytes follow different expressional kinetics during the 42-day *S. molnari* infection (**Figures 5H–J**). While both populations mounted a robust early response by 3 dpi, their further inflammatory trajectories diverged. In leukocytes, cytokine expression remained largely sustained, whereas *tnfα* levels steadily ebbed (**Figure 5H**; **Supplementary Figure 6B**). In contrast, erythrocytes emerged as active immunological sentinels, maintaining high *tnfα* and *ifnγ* levels throughout the infection, while other cytokines showed only a late-phase decline (**Figure 5H**; **Supplementary Figure 6B**). Pearson correlation uncovered a distinct wiring of the immune response as the infection transitioned from acute to the chronic infection phase (**Figure 5I**). We found that *ctslaA/B* and *ctsl.1B* in leukocytes were highly correlated within a unified inflammatory program during the acute phase, whereas *ctsl.1A* remained independent (**Figure 5I**, left). Erythrocytes, however, exhibited a more complex, phase-dependent expression pattern. The early co-expression of specific *ctsl* paralogs (*ctsl.1A/B, ctslaA*) with inflammatory markers (*ifnγ*, *tnfα*, *il-1β)* vanished at 28 dpi, only to resurge by 42 dpi with a new set of gene associations (**Figure 5I**, right). Comprehensive gene-gene correlation coefficients for all comparisons are provided in **Supplementary Figure 4**. This synchronized paralog switch underscores how different cell populations shift their proteolytic profiles as the infection landscape shifts (**Figure 5J**). Notably, in both leukocytes and erythrocytes, *ctsl.1A/B* showed a sharp decline at 28 dpi and remained transcriptionally suppressed at 42 dpi. This collapse aligns with the anticipated peak of parasite proliferation/presporogony in the blood (28 dpi) and liver (42 dpi) based on the established infection model (**Figure 5E**). This decline was mirrored by a synchronized induction of *ctslaA/B*, which reached peak expression in the chronic phase in both cell fractions (**Figure 5J**).

## 4 Discussion

Understanding how gene duplication shapes protease repertoires is central to decoding vertebrate immune and metabolic plasticity. Cathepsin L in polyploid teleosts provides a window into how evolutionary diversification can generate tissue- and cell-specific regulatory networks that balance host defense and homeostasis. To address this gap, we integrated evolutionary, genomic, and structural analyses to map the full *ctsl* repertoire in common carp, providing a framework to interpret how gene duplication drives tissue- and cell-specific regulation.

### 4.1 Ancient WGD and lineage-specific duplications shape the carp *ctsl* repertoire

Our phylogenetic analyses revealed that carp *ctsl* genes group into four main clades, *ctsl.1, cts12, ctsla*, and *ctsll*, with diversification events dating back to early vertebrates (1R, 2R) and the teleost ancestor (Ts3R) (Yu et al., 2024). Further expansion in carp is driven by Cs4R (Glasauer and Neuhauss, 2014; Xu et al., 2019), as evidenced by A/B ohnologs (paralogs arisen by WGD) in polyploid carp, goldfish, silver crucian carp, and barbels (Li et al., 2015; Braasch, 2020). Notably, our dataset indicates retention of both Ts3R-derived *ctsla* paralogs and subsequent divergence to the *ctsll* lineage in cyprinids, whereas the second *ctsla* paralog appears secondarily lost in several other clupeocephalan lineages, consistent with the well-known pattern of extensive, lineage-specific post-WGD gene loss. Such differential retention can be caused by neofunctionalization within the cyprinid lineage, a hypothesis supported by highly conserved local synteny. Our discovery of a novel *ctsl.1B’* paralog in carp and related cyprinids indicates an additional lineage-specific gene duplication, further enriching the *ctsl* gene repertoire. The observed collinearity suggests that while the *ctsl* family has undergone significant WGDs, the surrounding genomic neighborhoods remain evolutionary coldspots, likely due to functional constraints on the regulatory architecture of these proteolytic clusters (Simakov et al., 2022). Overall, carp *ctsl* genes have diversified through a combination of ancient WGDs and lineage-specific duplications, likely enabling sub- or neofunctionalization (Glasauer and Neuhauss, 2014; Li et al., 2015), as reflected in the distinct tissue-specific expression profiles of individual paralogs. At the same time, the co-expression of ohnologous pairs within the same clades (e.g., *ctsl.1A/B* and *ctslaA/B*) suggests that some duplicates may cooperate in particular tissues. This cooperation can potentially be executed via coordinated regulation, facilitated by the structural stability of the cluster (Li et al., 2015; Blasweiler et al., 2023), a pattern documented in other teleosts (Varadharajan et al., 2018). This combination of expression divergence and conserved genomic organization points to an evolutionary trajectory in which gene retention after duplication supports both functional specialization and overlapping roles, providing a validated baseline of the *ctsl* repertoire that potentially supports the metabolic and immunological plasticity of the species.

### 4.2 Carp CTSL paralogs diverge in the active-site cleft loops

Phylogeny-derived findings were corroborated by our structural and motif-based analyses, which resolved carp CTSL into four groups. Although all groups retain the conserved papain-like fold, they diverge in the hydrophobic and electrostatic profiles of the L1-L4 loops that shape the active-site cleft. In papain-like cysteine proteases, these loop elements sculpt the substrate-binding groove and delineate the S1–S4 and S1′–S4′ subsites, thereby establishing the local catalytic microenvironment and governing substrate specificity (Turk et al., 2000; Petushkova et al., 2022). Notably, subsite composition, particularly within the S2 pocket, a major determinant of specificity in cathepsin L-like proteases, can be reprogrammed by minimal residue changes, and swapping key S2-pocket residues has been shown to permute substrate preferences between cathepsins, underscoring how subtle loop/pocket remodeling can drive functional divergence (Lecaille et al., 2001, Lecaille et al., 2002). Consistent with this framework, the structural divergence we observe across the four CTSL groups likely translates into distinct substrate preferences and/or catalytic properties, providing a basis for their differential expression during the host response against the parasite. This interpretation aligns with broader evidence that cysteine cathepsins are tightly regulated in immune contexts and can be differentially deployed to support specialized roles in host defense and antigen-processing pathways (Nakagawa et al., 1998; Hsieh et al., 2002; Turk et al., 2012).

### 4.3 Common carp shows spatial distribution of *ctsl* paralogs

The common carp maintains a modular *ctsl* repertoire, with specific paralogs allocated to distinct tissues and cell types. Our high-resolution mapping shows that *ctsl.1A/B* are primarily enriched at mucosal interfaces like the gills and intestine. This distribution aligns with profiles in other teleosts (Whang et al., 2011; Li et al., 2015; Liang et al., 2017) and suggests these enzymes are specialized for epithelial homeostasis and frontline surveillance (Chen et al., 2020). In contrast, the *ctslaA/B* clade is predominant in systemic and metabolic tissues and is the only paralog expressed in circulating blood and head kidney leukocytes. Given the absence of other paralogs in immune cells, this indicates evolutionary specialization rather than redundancy of lysosomal proteases for distinct immune cell roles (Reiser et al., 2010). This spatial segregation is strengthened by mutually exclusive expression of *cts12* and *ctsll* paralogs, which remain entirely distinct from other cathepsins in steady-state tissues. Collectively, these patterns of pronounced expression divergence support a model where gene duplication serves as more than just a genomic backup. Instead, these patterns establish a baseline profile suggesting that the carp may utilize precision regulatory control to fulfill specialized, tissue/cell-restricted roles (Langham et al., 2004; Star et al., 2011; Glasauer and Neuhauss, 2014; Bermejo-Nogales et al., 2015; Lien et al., 2016). Alternatively, these patterns likely reflect either subgenome plasticity (Chen et al., 2024) or ontogenetic shifts (Tingaud-Sequeira and Cerdà, 2007), where biased homoeolog expression allows the carp to tailor its protease toolkit for both larval development and ecological resilience.

### 4.4 Infection activates an inflammation-independent *ctsl* transcriptional program

*S. molnari* infection triggers a spatiotemporal mismatch between the *ctsl* landscape and canonical inflammatory signaling. Although cytokines typically drive cathepsin modulation (Gerber et al., 2001; Fløyel et al., 2021), the acute induction of *tnfα*, *il-1β*, and *ifnγ* coincided with a systemic silencing of all examined *ctsl* paralogs. Notably, the strongest suppression affected *ctsl.1A/B* in major lymphoid tissues (spleen, head kidney, and leukocytes), which represent key sites of lysosomal antigen processing and MHC class II peptide loading (Roche and Furuta, 2015). Transient downregulation of these paralogs during peak cytokine induction may therefore impair antigen processing and immune surveillance. Comparable immune-evasion strategies have been reported in other parasite systems, including interference with host MHC II processing by *Leishmania* spp. (De Souza Leao et al., 1995; Podinovskaia and Descoteaux, 2015) and suppression of host cathepsin activity during helminth infections (Hartmann and Lucius, 2003). This inverse relationship suggests a potential functional trade-off, where the system transiently prioritizes immediate innate defense at the expense of lysosomal proteolysis, independently of classical cytokine signaling (Nakagawa and Rudensky, 1999; Müller et al., 2012; Watts, 2012). Recovery of *ctsl* expression by 7 dpi, suggests a restored proteolytic balance. At 28–42 dpi, inflammatory gene expression fluctuated independently from this balanced *ctsl* transcription. These dynamics match host responses during *S. molnari* infection, with moderate early inflammation, peak lymphocyte activity around 28 dpi, and dominant anti−inflammatory and humoral responses by 35 dpi (Korytář et al., 2019; Korytář et al., 2020). Furthermore, such patterns also position the lysosome as a central sensing immune hub (Bonacina et al., 2025; Jain and Zoncu, 2026) that may respond independently of the broader cytokine activation. We speculate this early *ctsl* suppression reflects a stealth phase in which the parasite attenuates antigen processing and MHC class II loading to evade detection (Villadangos and Ploegh, 2000; Leroux et al., 2015; Roche and Furuta, 2015). Our study provides a baseline validation of this transcriptional asynchrony. Whether this reflects a specific parasitic strategy or a broader host response remains to be resolved by future functional studies.

### 4.5 *ctsl* transcription shows cell- and temporal reprogramming during immune response

Effective immunity requires a delicate balance between pathogen clearance and tissue homeostasis (Rivera et al., 2016; Li et al., 2025). Our model reveals that carp leukocytes and erythrocytes achieve this balance through divergent transcriptional programs. While leukocyte *ctslaA/B* and *ctsl.1B* remain tightly integrated into canonical inflammatory networks, erythrocytic *ctsl* display a distinct temporal dissociation (Creasy and McCoy, 2011). This suggests that nucleated teleost erythrocytes, increasingly recognized as active immunological players (Shen et al., 2018; Stosik et al., 2020; Chan et al., 2023; Majstorović et al., 2024), may repurpose *ctsl* proteolysis to mitigate infection-induced oxidative stress and metabolic shifts caused by circulating parasites. Although multiple studies report *ctsl* orthologs as infection-responsive in teleost tissues (Sun and Hu, 2015; Wang et al., 2015; Chen et al., 2020; Li et al., 2022; Esteban, 2024), their cell-specific regulation has remained an enigma. Our findings provide a baseline mapping of transcriptional realignment of *ctsl* transcription, positioning the lysosome as a multifaceted sensing hub governed strictly by cellular context (Conus, 2010; Ballabio and Bonifacino, 2020). Beyond the lysosome, cathepsin L may localize to the cell surface (Ahimbisibwe et al., 2010) or be secreted to reshape the extracellular landscape or modulate immune challenge through the proteolytic mechanisms (Collette et al., 2004; Creasy and McCoy, 2011; Yadati et al., 2020; Anes et al., 2022; Zhao et al., 2024). Hence, further functional studies are required to delineate how these cell-specific transcriptional shifts translate into proteomic and immune outcomes.

## 5 Conclusion

The common carp *ctsl* gene family has expanded through ancient WGD and lineage-specific duplications, generating paralogs with structurally conserved folds but divergent substrate-binding loops. These paralogs display pronounced tissue- and cell-specific expression, with erythrocytes deploying noncanonical transcriptional programs independent of systemic cytokine cues. During *S. molnari* infection, *ctsl* regulation is temporally realigned, uncoupled from inflammatory signaling, revealing a flexible proteolytic network. Collectively, these findings uncover a previously unrecognized layer of immune and metabolic specialization mediated by duplicated lysosomal proteases in polyploid teleosts.

Future work should test whether the paralog-resolved transcriptional shifts reported here translate into measurable changes in cathepsin L protein abundance and enzymatic activity during infection. Key next steps include infection time-course sampling optimized for protein preservation, combined with (i) bulk cathepsin activity assays under class-specific inhibitor control and activity-based probe profiling to quantify the active cysteine cathepsin pool, and (ii) targeted proteomics and/or development of paralog-discriminating reagents (e.g., paralog-specific peptides, antibodies, or engineered substrates) to resolve contributions of individual *ctsl* paralogs. Finally, gene-specific perturbation approaches (e.g., *ex vivo* manipulation of primary carp cells or emerging *in vivo* knockdown/editing strategies) will be essential to causally link selected paralogs to immune functions, erythrocyte remodeling, and infection outcomes.

## Data Availability

The datasets presented in this study can be found in online repositories. The names of the repository/repositories and accession number(s) can be found in the article/**Supplementary Material**.
