# Supplementary material for: Evolutionary diversification and immunoprofiling of cathepsin L toolkit in common carp
Source: Front Cell Infect Microbiol. 2026 Apr 7;16:1805838. doi: 10.3389/fcimb.2026.1805838 (PMC13095802; doi:10.3389/fcimb.2026.1805838)
Supplement: Supplementary file 1 [file SupplementaryFile1.zip › Supplementary Table 1.docx]

**Supplementary Table 1.** List of *Cyprinus carpio* genomes used for the identification of *ctsl* genes.

| **Common carp strain** | **Genomic assembly/WGS** | **Reference** |
| --- | --- | --- |
| European common carp strain R3xR8 | GCA_001270105.1/LHQP01 | Kolder et al., 2016 |
| European common carp strain R3xR8 | GCA_905221575.1/CAJNDQ01 | Blasweiler et al., 2023 |
| Chinese common carp strain Songpu | GCA_000951615.1/- | Xu et al., 2014 |
| Chinese common carp strain Songpu | GCA_018340385.1/JAEOAB01 | Li et al., 2021 |
| German mirror carp | GCA_004011555/SAUK01 | Xu et al., 2019 |
| Wuyuan Hebao red carp | GCA_004011595/SAUJ01 | Xu et al., 2019 |
| Yuxuan Yellow River (Huanghe) carp | GCA_004011575/SAUI01 | Xu et al., 2019 |
