# Supplementary material for: Evolutionary diversification and immunoprofiling of cathepsin L toolkit in common carp
Source: Front Cell Infect Microbiol. 2026 Apr 7;16:1805838. doi: 10.3389/fcimb.2026.1805838 (PMC13095802; doi:10.3389/fcimb.2026.1805838)
Supplement: Supplementary file 1 [file SupplementaryFile1.zip › Supplementary Table 3.docx]

| **Supplementary Table 3.** List of primers used for PCR validation of *in silico* identified common carp *ctsl* genes. | | | |
| --- | --- | --- | --- |
| **Gene** | **Forward and reverse primer pair name: sequence** | **Anneal. temp. (°C)** | **Amplicon length (bp)** |
| *ctsl.1A* | G1O2FULLf_NEW: 5'-TAA GGA AAC ATA TAT CCT ATG AAC-3';  G1O2FULLr_NEW: 5'-AAC CAC GGA GGT CCA CGT TGA C-3' | 52 | 1068 |
| *ctsl.1B,*  *ctsl.1B'* | G1O1FULLf_NEW: 5'-CAA GGA AAC GTA TAT CCT GAA TAC-3';  G1O1FULLr_NEW: 5'-TTC CAC TCC AGG TCC ATG TTC AT-3' | 53 | 1078 |
| *cts12A* | 1st round PCR:  G2O2Fullf_NEW: 5'-GGA CGT ATC AAG CAC ACT GGG C-3';  G2OallFULLrev: 5'-TTA GAC CAG AGG GTA ACT G-3’ | 55 | 1032 |
|  | 2nd round PCR:  CathL_Cc_G2O2f2: 5'-ATC TCC ATC AAA TAT CCT ACA ATG AT-3';  CathL_Cc_G2O2r2out: 5'-GCA AGT GTT TTT ACC ATT GCG G-3' | 55 | 772 |
| *cts12B* | 1st round PCR:  G2O1Fullf_NEW: 5'-ATA CGT GTC AAG CAC ACT GGG T-3';  G2OallFULLrev: 5'-TTA GAC CAG AGG GTA ACT G-3’ | 55 | 926 |
|  | 2nd round PCR:  CathL_Cc_G2O1f2: 5'-CTC AGA GTG GGC TTT GTG GAA AAG A-3';  CathL_Cc_G2O1r2: 5'-GCT GTA GAA GAG GAA GCT TGG ATG A-3' | 55 | 727 |
| *ctsllA* | 1st round PCR:  CathL_Cc_G3O1f2out: 5'-TGC TCT TCA CAC TAT GCT TGA GTG-3';  CathL_Cc_G3O1r2out: 5'-CGC AAT GCC ACA ATG GTT CTG-3' | 60 | 962 |
|  | 2nd round PCR:  CathL_Cc_G3O1f2in: 5'-TTC TTC ACT GCT GCA CTC CTC G-3';  CathL_Cc_G3O1r2in: 5'-CTT AAA CCA GCA GTT AGA TGA TCA CTG-3' | 60 | 763 |
| *ctsllB* | 1st round PCR:  CathL_Cc_G3O2f2out: 5'-CAT TCC TCA CTC TAT GCT TGA GTG C-3';  CathL_Cc_G3O2r2out: 5'-ATG TTG CAA TGC CAC AAT GGT TTT C-3' | 60 | 975 |
|  | 2nd round PCR:  CathL_Cc_G3O2f2in: 5'-ATT AAA CCT GCA TTT AGA TGA TCA CTG-3';  CathL_Cc_G3O2r2in: 5'-GAC TGC ATG GTC AAG ATC TTC AG-3' | 58 | 787 |
| *ctslaA* | G4O1FULLf_NEW: 5'-AGC CTT TCT GCA ACA CAC CGT-3';  G4O1FULLr_NEW: 5'-GAC ACT AAT GTG GCC TTC TGG TAC C-3' | 59 | 1123 |
| *ctslaB* | G4O2FULLf_NEW: 5'-CGC GCT ATA AAC CTG GGG CCG C-3';  G4O2FULLr_NEW2: 5'-TTA CCA CTA ATG TGA TCT TCT GCT ACT-3' | 60 | 1105 |
