# Supplementary material for: Evolutionary diversification and immunoprofiling of cathepsin L toolkit in common carp
Source: Front Cell Infect Microbiol. 2026 Apr 7;16:1805838. doi: 10.3389/fcimb.2026.1805838 (PMC13095802; doi:10.3389/fcimb.2026.1805838)
Supplement: Supplementary file 1 [file SupplementaryFile1.zip › Supplementary Table 4.docx]

**Supplementary Table 4.** List of qPCR primers used to amplify common carp *ctsl* genes.

| **Gene name** | **Forward and reverse primer pair name: sequence** | **Amplicon length (bp)** |
| --- | --- | --- |
| *ctsl.1A* | CathL_Cc_G1O2_qPCRF: 5'-GCG TTC CAT GGA TGC CTG G-3';  CathL_Cc_G1O2_qPCRR:5'-TCG GTC ACA TAG CCC TTG TCC-3';  CathL_Cc_G1O2_probe: 5'-HEX-AGG ATG CCG TTG TGA TTC CCA GTT C-BHQ1-3' | 128 |
| *ctsl.1B* | CathL_Cc_G1O1_qPCRF: 5'-GGT GGA CTG CTC TGG TTC TTA-3';  CathL_Cc_G1O1_qPCRR: 5'-CAG TGC TCG GGT TAA AAC GG-3';  CathL_Cc_G1O1_probe: 5'-HEX-TGT GGT GGA GGA CTA ATG GAC CAG-BHQ1-3' | 155 |
| *ctslaA* | CathL_Cc_G4O1_qPCRF: 5'-GCT ATT GAC GCT GGA CAC GA-3';  CathL_Cc_G4O1_qPCRR: 5'-TGT AGA TGT ATC CTT TAT CAC CCC AG-3';  CathL_Cc_G4O1_probe: 5'-HEX-AGG AGC TAG ATC ATG GCG TCC TTG C-BHQ1-3' | 199 |
| *ctslaB* | CathL_Cc_G4O2_qPCRF: 5'-TCA TGG TGT GCT TGC CGT TG-3';  CathL_Cc_G4O2_qPCRR: 5'-TTA GAC GAG AGG GTA GCT AGC AG-3';  CathL_Cc_G4O2_probe: 5'-HEX-TTG GGG TGA TAA AGG TTA TGT CTA CAT GG-BHQ1-3' | 178 |
| *β-actin* | Actin_Cc_DNA_F: 5'-AGG TAT GGA GTC TTG CGG TA-3';  Actin_Cc_DNA_R: 5’-ACA GGT CCT TAC GGA TGT CG-3';  Actin_Cc_DNA_P: 5'-FAM-TGAGACCACCTTCAACTCCAT-BHQ1-3’ | 80 |
