# Supplementary material for: Evolutionary diversification and immunoprofiling of cathepsin L toolkit in common carp
Source: Front Cell Infect Microbiol. 2026 Apr 7;16:1805838. doi: 10.3389/fcimb.2026.1805838 (PMC13095802; doi:10.3389/fcimb.2026.1805838)
Supplement: Supplementary file 1 [file SupplementaryFile1.zip › Supplementary Table 5.docx]

**Supplementary Table 5.** List of qPCR primers used to amplify common carp cytokine genes.

| **Gene** | **Oligo sequence (5’- 3’)** | **Primer efficiency (%)** | **Reference** |
| --- | --- | --- | --- |
| *ef1-α* | FW: GACAACCCCAAGGCTCTCAA  RV: ACAGCAAAGCGACCAAGAGG | 97.3 | (Yamaguchi et al., 2013) |
| *il6* | FW: CAGATAGCGGACGGAGGGGC  RV: GCGGGTCTCTTCGTGTCTT | 94.3 | (Baloch et al., 2023) |
| *il-1β* | FW: AAGGAGGCCAGTGGCTCTGT  RV: CCTGAAGAAGAGGAGGCTGTCA | 97.5 | (Baloch et al., 2023) |
| *tnfα* | FW: GCTGTCTGCTTCACGCTCAA  RV: CCTTGGAAGTGACATTTGCTTTT | 94.9 | (Ribeiro et al., 2010) |
| *ifnγ* | FW: CGATCAAGGAAGATGACCCAGTC  RV: GTTGCTTCTCTGTAGACACGCTTC | 98.4 | (Embregts et al., 2019) |
| *il10* | FW: CGCCAGCATAAAGAACTCGT  RV: TGCCAAATACTGCTCGATGT | 97.9 | (Baloch et al., 2023) |

References:

1. Baloch, A. A., Steinhagen, D., Gela, D., Kocour, M., Piačková, V., & Adamek, M. (2023). Immune responses in carp strains with different susceptibility to carp edema virus disease. *PeerJ*, *11*, e15614. https://doi.org/10.7717/peerj.15614
2. Embregts, C. W. E., Tadmor-Levi, R., Veselý, T., Pokorová, D., David, L., Wiegertjes, G. F., & Forlenza, M. (2019). Intra-muscular and oral vaccination using a Koi Herpesvirus ORF25 DNA vaccine does not confer protection in common carp (*Cyprinus carpio* L.). *Fish & Shellfish Immunology*, *85*, 90–98. https://doi.org/10.1016/j.fsi.2018.03.037
3. Ribeiro, C. M. S., Pontes, M. J. S. L., Bird, S., Chadzinska, M., Scheer, M., Verburg-van Kemenade, B. M. L., Savelkoul, H. F. J., & Wiegertjes, G. F. (2010). Trypanosomiasis-Induced Th17-Like Immune Responses in Carp. *PLoS ONE*, *5*(9), e13012. https://doi.org/10.1371/journal.pone.0013012
4. Yamaguchi T, Katakura F, Someya K, Dijkstra JM, Moritomo T, Nakanishi T. Clonal growth of carp (*Cyprinus carpio*) T cells in vitro: long-term proliferation of Th2-like cells. Fish & shellfish immunology. 2013 Feb 1;34(2):433-42.
