# Supplementary figures and images for: Evolutionary diversification and immunoprofiling of cathepsin L toolkit in common carp
Source: Front Cell Infect Microbiol. 2026 Apr 7;16:1805838. doi: 10.3389/fcimb.2026.1805838 (PMC13095802; doi:10.3389/fcimb.2026.1805838)

A

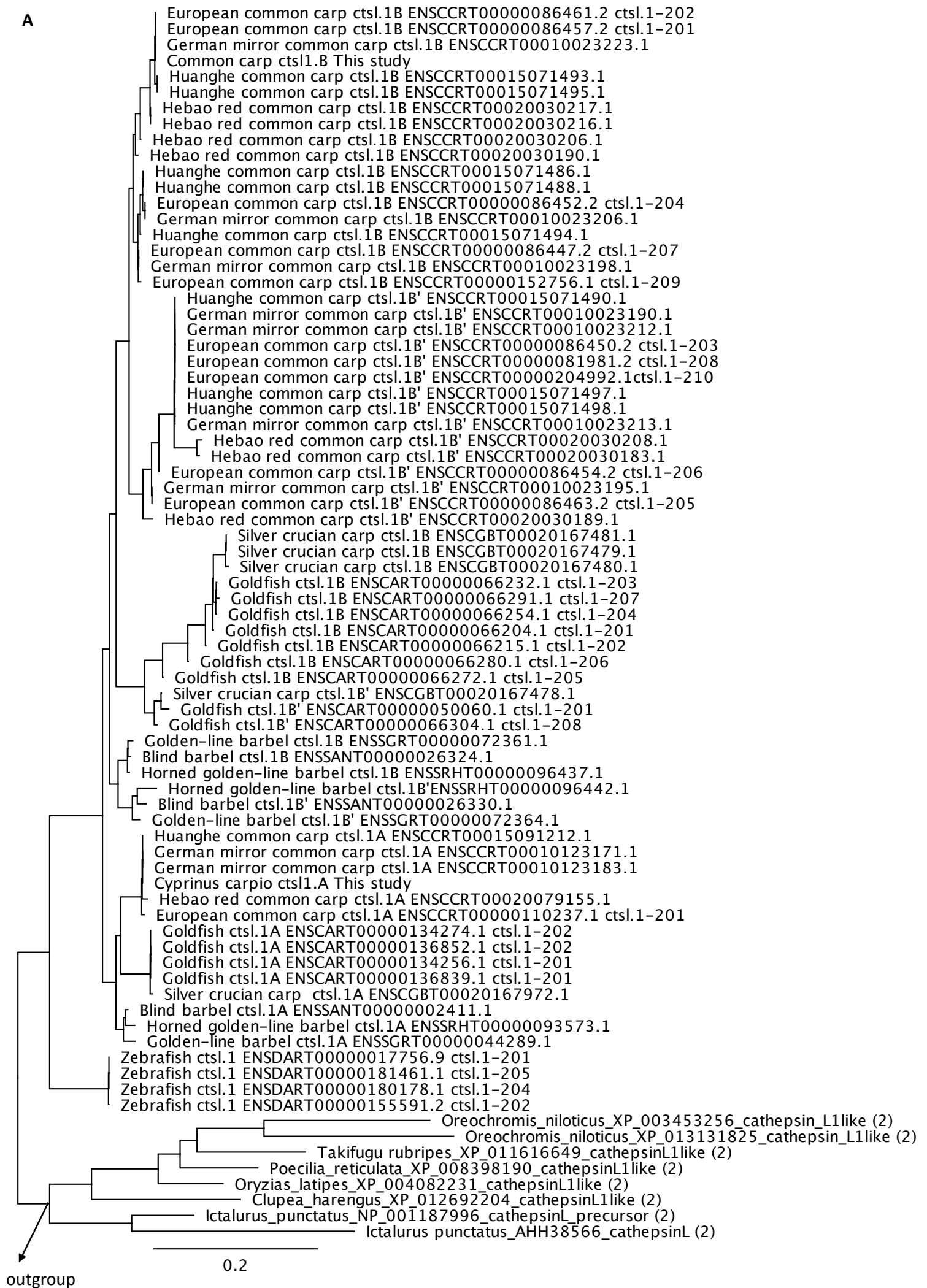

B

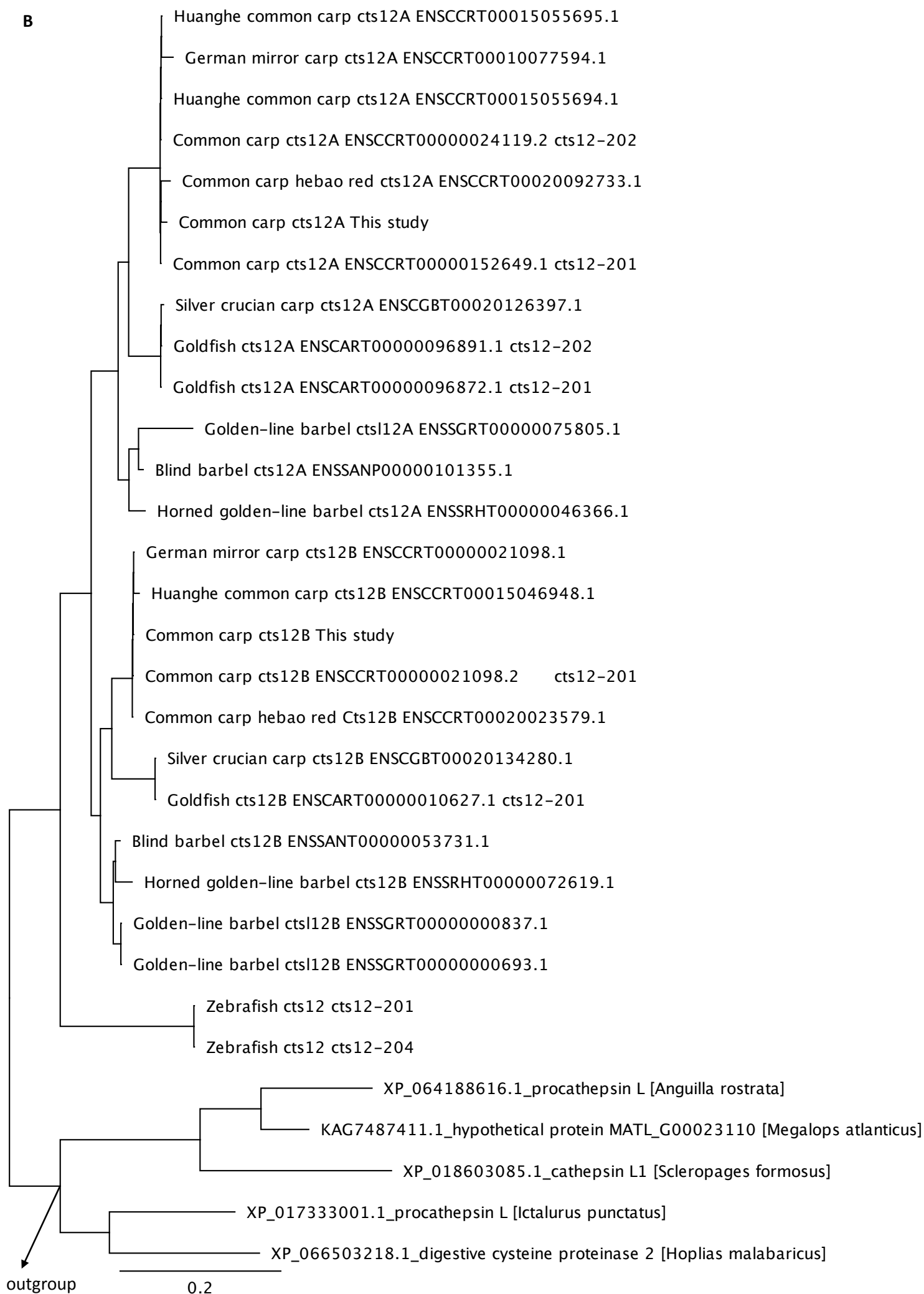

c

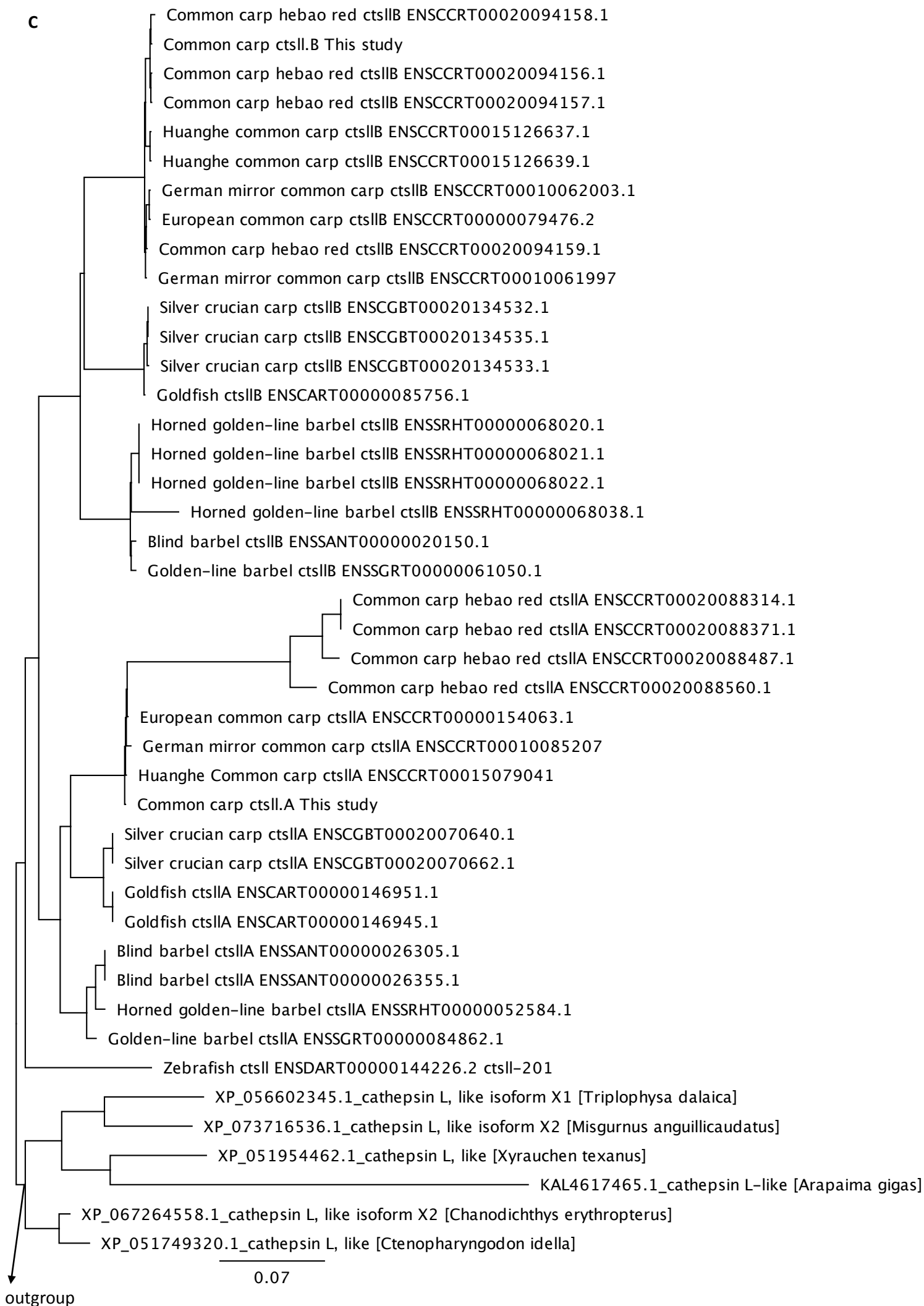

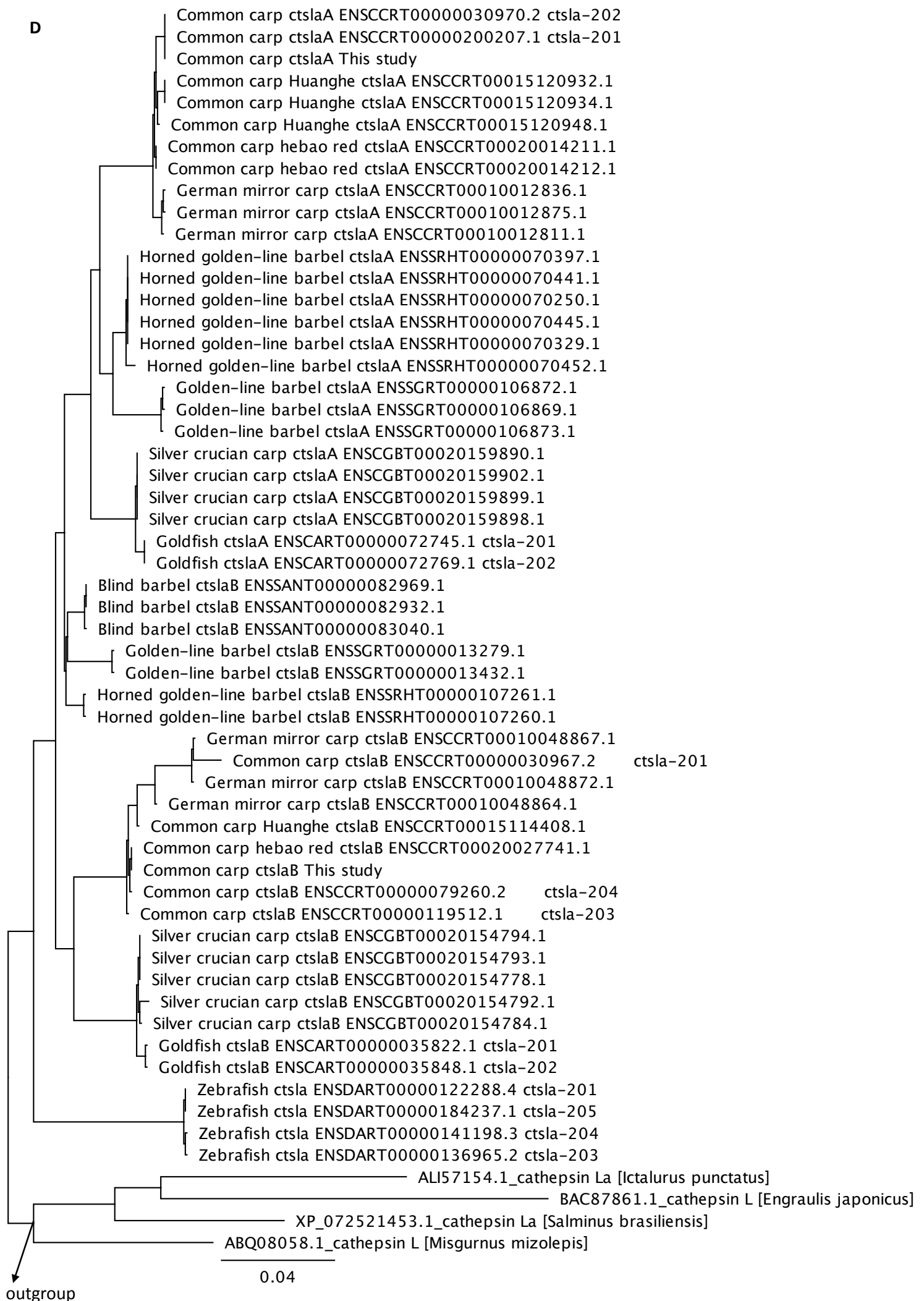

Supplement: Supplementary file 1 [file SupplementaryFile1.zip › Supplementary Figure 2.pdf]

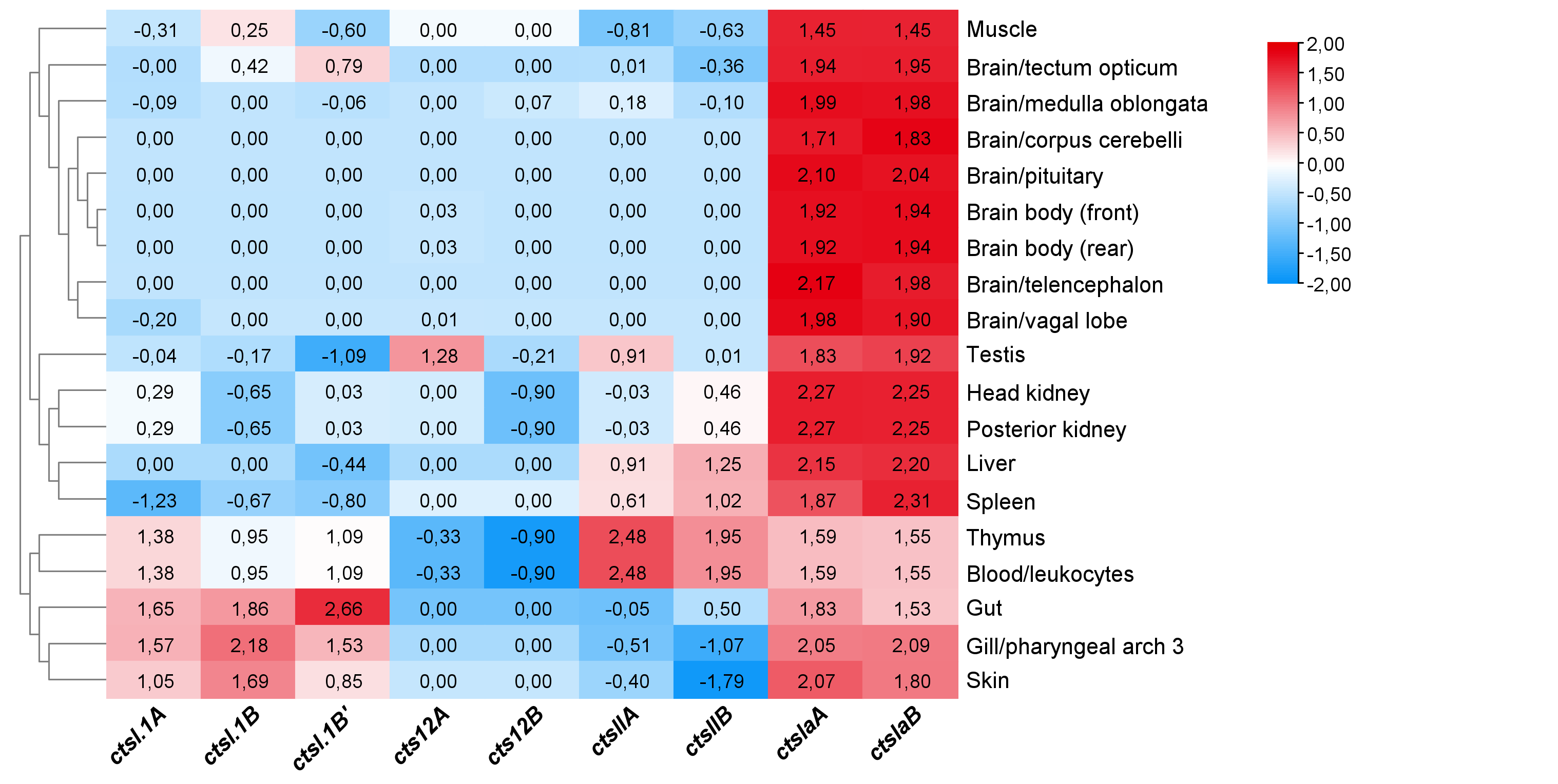

Supplement: Supplementary file 1 [file SupplementaryFile1.zip › Supplementary Figure 3.tiff]

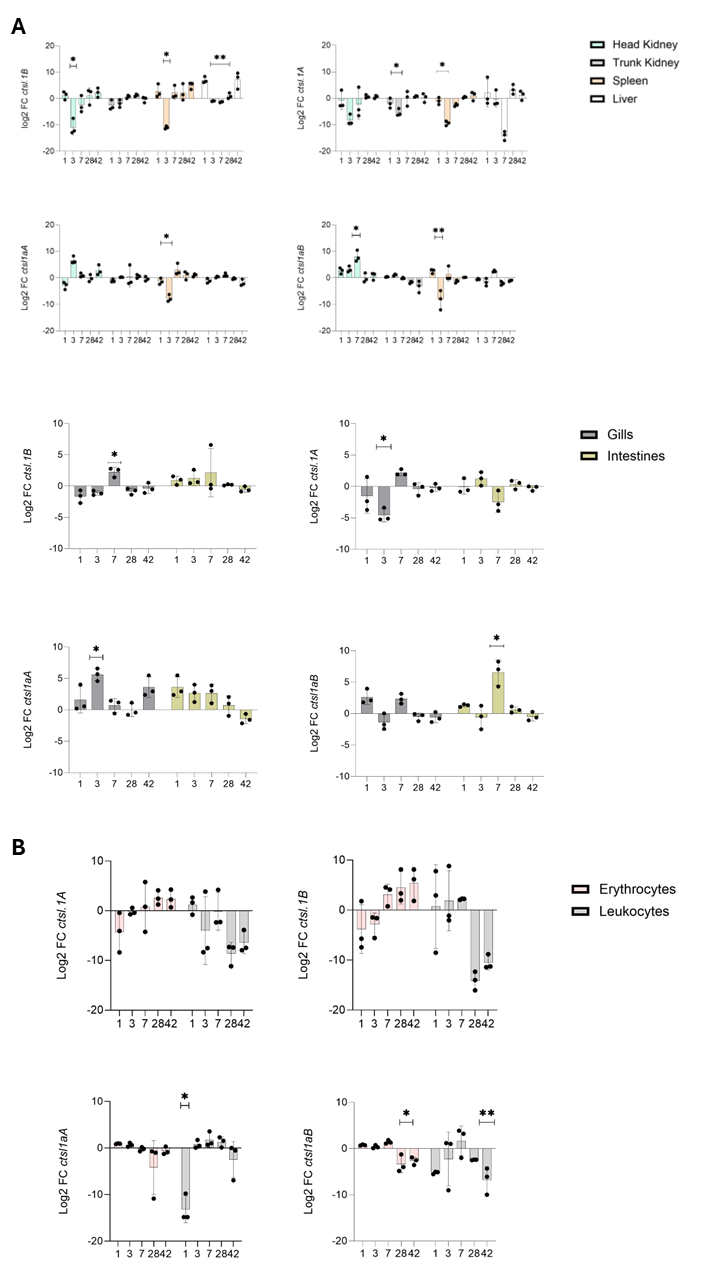

Supplement: Supplementary file 1 [file SupplementaryFile1.zip › Supplementary Figure 5.tif]

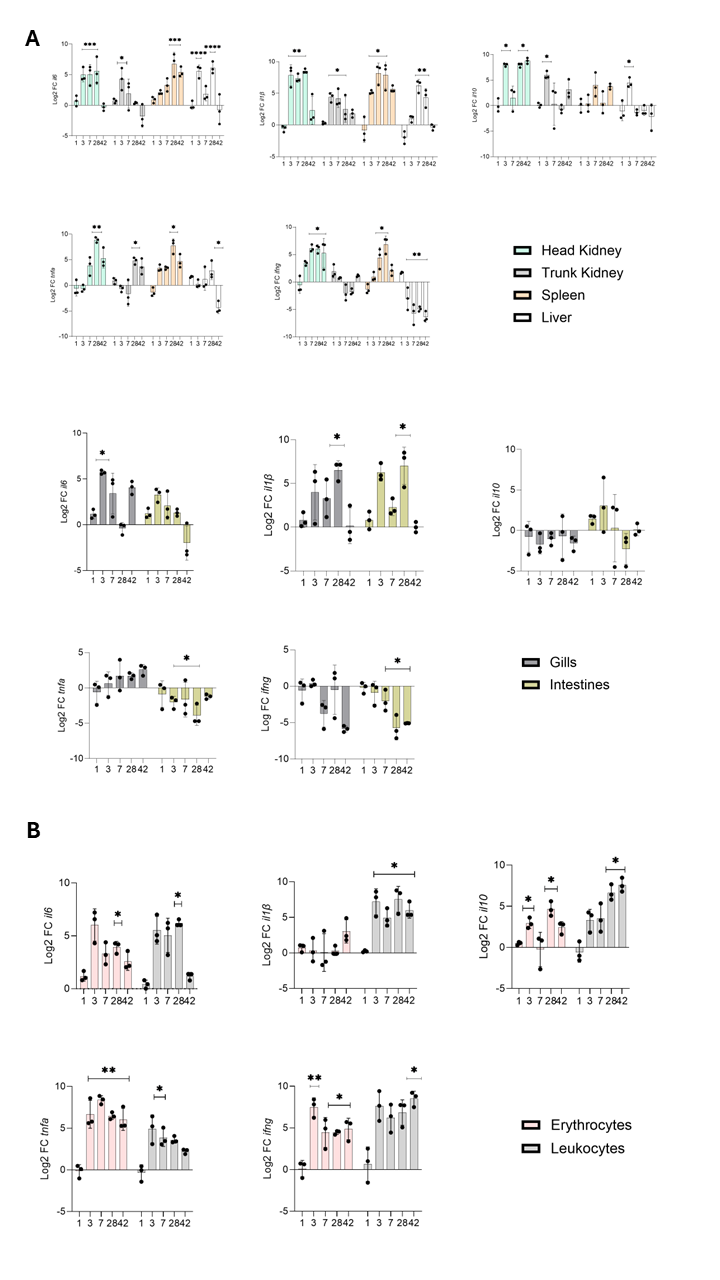

Supplement: Supplementary file 1 [file SupplementaryFile1.zip › Supplementary Figure 6.tif]
